# Trends in the prevalence of metabolic syndrome and its components in South Korea: Findings from the Korean National Health Insurance Service Database (2009–2013)

**DOI:** 10.1371/journal.pone.0194490

**Published:** 2018-03-22

**Authors:** Seung Eun Lee, Kyungdo Han, Yu Mi Kang, Seon-Ok Kim, Yun Kyung Cho, Kyung Soo Ko, Joong-Yeol Park, Ki-Up Lee, Eun Hee Koh, on Behalf of the Taskforce Team of Diabetes Fact Sheet of the Korean Diabetes Association

**Affiliations:** 1 Department of Internal Medicine, Asan Medical Center, University of Ulsan College of Medicine, Seoul, Korea; 2 Department of Biostatistics, The Catholic University of Korea, Seoul, Korea; 3 Department of Clinical Epidemiology and Biostatistics, Asan Medical Center, University of Ulsan College of Medicine, Seoul, Korea; 4 Department of Internal Medicine, Cardiovascular and Metabolic Disease Center, Inje University Sanggye Paik Hospital, Inje University College of Medicine, Seoul, Korea; Beijing Key Laboratory of Diabetes Prevention and Research, CHINA

## Abstract

**Background:**

The prevalence of metabolic syndrome has markedly increased worldwide. However, studies in the United States show that it has remained stable or slightly declined in recent years. Whether this applies to other countries is presently unclear.

**Objectives:**

We examined the trends in the prevalence of metabolic syndrome and its components in Korea.

**Methods:**

The prevalence of metabolic syndrome and its components was estimated in adults aged >30 years from the Korean National Health Insurance Service data from 2009 to 2013. The revised National Cholesterol Education Program criteria were used to define metabolic syndrome.

**Results:**

Approximately 10 million individuals were analyzed annually. The age-adjusted prevalence of metabolic syndrome increased from 28.84% to 30.52%, and the increasing trend was more prominent in men. Prevalence of hypertriglyceridemia, low HDL-cholesterol, and impaired fasting plasma glucose significantly increased. However, the prevalence of hypertension decreased in both genders. The prevalence of abdominal obesity decreased in women over 50 years-of-age but significantly increased in young women and men (<50 years).

**Conclusions:**

The prevalence of metabolic syndrome is still increasing in Korea. Trends in each component of metabolic syndrome are disparate according to the gender, or age groups. Notably, abdominal obesity among young adults increased significantly; thus, interventional strategies should be implemented particularly for this age group.

## Introduction

Metabolic syndrome is a constellation of cardiovascular risk factors, including hypertension, central obesity, dyslipidemia, and hyperglycemia. Individuals with metabolic syndrome are more prone to developing type 2 diabetes [1] and are at a greater risk for cardiovascular and all-cause mortality [2, 3].

During the past several decades, the prevalence of metabolic syndrome has markedly increased worldwide [4]. Behavioral and environmental changes, such as adoption of a westernized diet and sedentary lifestyle following the socioeconomic rise in developing countries, are thought to be the main reasons for this pandemic of metabolic syndrome [5]. Likewise, South Korea experienced a rapid socioeconomic growth during the 20^th^ century and the national income has been rising until the present [6]. Subsequently, Korean individuals have reported decreased physical activity and increased consumption of meals with greater proportions of meat, dietary fat [7] and simple sugars [8], leading to an increase in overall body weight. Accordingly, recent reports indicated that the prevalence of diabetes and mortality from cardiovascular disease (CVD), which represent clinical outcomes of metabolic syndrome, has also increased in Korea [9, 10]. Interestingly, studies in the United States found that the prevalence of metabolic syndrome remained stable or slightly declined in recent years, possibly due to increased awareness of metabolic syndrome and its health consequences [11, 12]. However, it is uncertain whether this change in the trend of metabolic syndrome is also the case in other countries. Although the mass media and government have made various efforts recently to prevent further increases in metabolic diseases in Korea, the effectiveness of these efforts for prevention of metabolic syndrome is unclear.

Insulin resistance is generally regarded as a single common etiology of each component of metabolic syndrome [13, 14]. However, several previous studies using cluster analysis revealed that multiple independent factors may contribute to metabolic syndrome, and that not all components of metabolic syndrome can be grouped by a single etiologic factor [15, 16]. Thus, investigating the trends in each component of metabolic syndrome is important. In this study, we aimed to evaluate the temporal trends of metabolic syndrome and its components in Korea using data from a large population-based survey. Furthermore, we performed subgroup analyses stratified by gender, age, or presence of impaired fasting glucose (IFG) to clarify the association with each component.

## Materials and methods

### Data source and study population

The Korean National Health Insurance Service, run by the government of South Korea, manages public healthcare at the national level. The detailed structure and function of Korean National Health Insurance Service is described elsewhere [17]. The Korean National Health Insurance Service manages all databases of Korea’s health service utilization. We used regular health check-up data, which include employee subscribers, regional insurance subscribers who are regional householders, household members aged >40 years, and medical aid beneficiaries [17]. We selected data from individuals ≥30 years old who did not have missing information between 2009 and 2013. The study design was approved by the Korean National Institute for Bioethics Policy (P01-201504-21-005).

### Clinical and laboratory measurements

All participants were required to complete self-administered questionnaires that included smoking and alcohol habits, physical activity, and past medical history. Smoking status was classified as current smoker or not. Heavy drinker was defined as consumption of >210 g alcohol per week. Participants who met one of the following criteria were defined as physically active: 1) ≥3 days of vigorous activity for ≥20 min/day or 2) ≥5 days of moderate intensity activity or walking for ≥30 min/day. Height, weight, waist circumference (WC), systolic blood pressure (BP), and diastolic BP were obtained by a trained nurse. Body mass index (BMI) was calculated as weight in kilograms divided by height in square meters (kg/m^2^). Venous blood sampling was performed after overnight fasting to determine fasting plasma glucose (FPG), total cholesterol, triglyceride (TG), high-density lipoprotein (HDL) cholesterol, low-density lipoprotein cholesterol, aspartate aminotransferase, alanine aminotransferase, gamma-glutamyl transferase, hemoglobin, and creatinine levels.

### Definitions of metabolic syndrome and IFG

Metabolic syndrome was defined using a revised National Cholesterol Education Program definition [18], which adopted an Asian-specific WC threshold suggested by the International Diabetes Foundation [19]. Individuals with three or more of the following criteria were defined as having metabolic syndrome:

1) Abdominal obesity; WC ≥90 cm in men or ≥80 cm in women;
2) Hypertriglyceridemia; TG ≥150 mg/dL or medication use;
3) Low HDL-cholesterol; HDL-cholesterol <40 mg/dL in men and <50 mg/dL in women or medication use;
4) High BP; systolic BP ≥130 mmHg and/or diastolic BP ≥85 mmHg or use of antihypertensive agents;
5) Hyperglycemia; FPG ≥100 mg/dL or use of antidiabetic medication.

IFG was defined in accordance with the 2017 American Diabetes Association criteria as FPG between 100 and 125 mg/dL. When comparing the prevalence of metabolic syndrome between individuals with or without IFG, we defined metabolic syndrome as the presence of two or more of the four components stated above 1)−4) to avoid colinearity between IFG and 5) hyperglycemia.

### Statistical analysis

To estimate the increase in rate over the 4 year period, age-adjusted prevalence of metabolic syndrome was determined by direct adjustment standardization method. We used the general linear model to examine the trends of metabolic syndrome and its components. Subgroup analysis was performed after classifying the participants according to sex, age, or status of glucose metabolism (normal vs. IFG). We analyzed the interaction P-value to assess for gender differences in metabolic syndrome trends between men and women. Logistic regression was used to examine the prevalence odds ratio (OR) per year between 2009 and 2013. To display and compare the prevalence among six age groups, we used forest plots with 95% Confidence intervals (CIs) [20]. Furthermore, subgroup-specific analysis was performed to determine the risk for metabolic syndrome according to IFG status. Statistical analyses were achieved using the SAS survey procedure (version 9.2; SAS Institute, Cary, NC).

## Results

The characteristics of Korean adults who participated in a regular health check-up between 2009 and 2013 are presented in Table 1. The data consisted of approximately 10 million individuals per year, which was equivalent to about 20% of the Korean population.

**Table 1:** Characteristics of Korean adults who participated in regular health check-ups, between 2009 and 2013.

			Year					*P*	
			2009	2010	2011	2012	2013		
Number of participants			9,069,127	10,135,257	10,399,685	10,974,490	10,583,081		
Age (years) ^a^	50.1 (12.5)		50.1 (12.5)	50.2 (12.6)	50.5 (12.5)	50.8 (12.7)		<0.01	
Men (%)	55.14		55.18	54.99	54.61	54.53		<0.01	
BMI (kg/m^2^) ^a^	23.9 (3.1)		23.9 (3.2)	23.9 (3.2)	23.9 (3.2)	23.9 (3.3)		<0.01	
WC (cm) ^a^	81.0 (8.8)		80.8 (8.9)	80.9 (8.9)	80.8 (9)	81 (9.1)		<0.01	
Physically active subjects (%)	22.96		23.59	23.78	24.59	25.41		<0.01	
Current smoker, n(%)	25.18		24.47	24.75	24.02	24.11		<0.01	
Heavy drinker, n(%)	7.68		7.58	7.49	7.27	7.22		<0.01	

BMI, body mass index; WC, waist circumference.

^a^Age, BMI, and WC were expressed as mean (SD).

**Fig 1:**
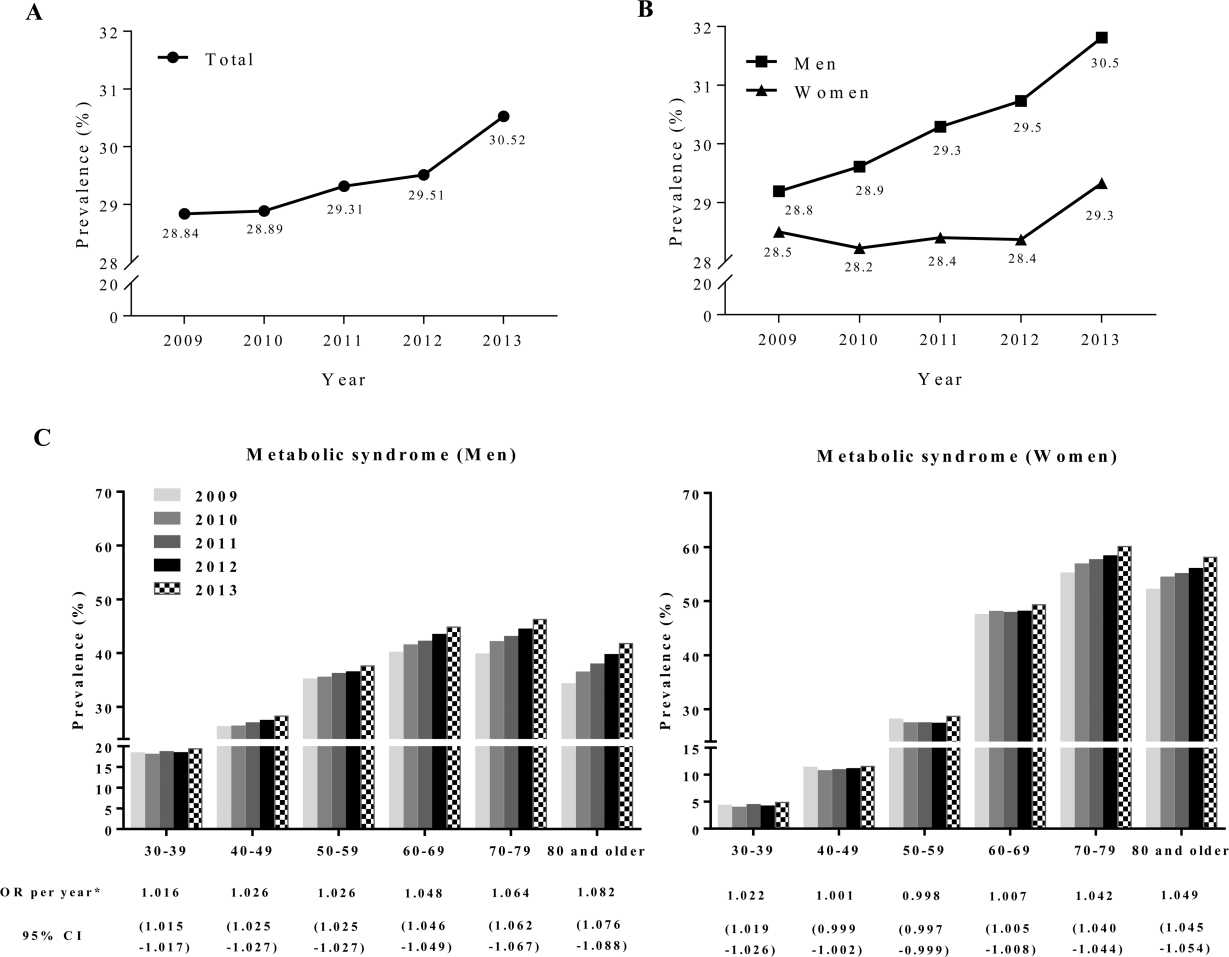
Trends in metabolic syndrome in Korea. The trends in metabolic syndrome prevalence among Korean adults, who participated in regular health check-ups between 2009 and 2013. *A*: Overall population (*P* for trend <0.01). *B*: Comparison between men and women (*P* <0.001). *C*: Age-specific trends of metabolic syndrome and ORs per year between 2009 and 2013. ^*^Age-stratified logistic regression was used for the calculation of OR per year. OR = Odds ratio.

Over the course of the survey, the age-adjusted prevalence of metabolic syndrome increased significantly from 28.84% in 2009 to 30.52% in 2013 (*P* <0.01) (Fig 1A). The absolute change was 1.68% over the 4-year period, thereby making the annual increase approximately 0.4%. This increase occurred in both genders but was more pronounced in men than in women (*P* < 0.001) (Fig 1B).

To investigate the associations between age and the trends of metabolic syndrome, we performed age-stratified analysis (Fig 1C). In both genders, the increase in metabolic syndrome was prominent in elder individuals. For example, in individuals ≥80 years old, the prevalence of metabolic syndrome increased from 34.37% to 41.88% in men and from 52.29% to 58.21% in women.

**Fig 2:**
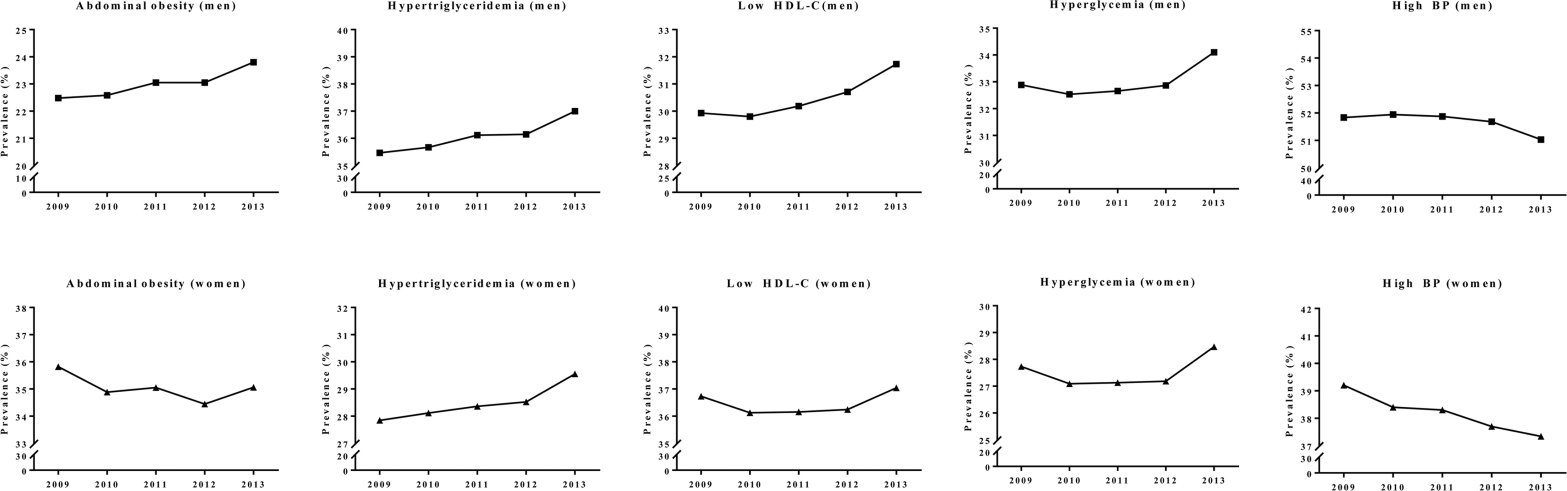
The trends of each component of metabolic syndrome. The trends of each component of metabolic syndrome in individuals stratified by gender. *P* for trends were all significant (*P* <0.01). BP = blood pressure, HDL = high-density lipoprotein.

Trends in the components of metabolic syndrome differed between genders (Fig 2). The prevalence of abdominal obesity increased significantly in men (from 22.48% in 2009 to 23.80% in 2013; net increase of 1.32%), but decreased in women (from 35.82% to 35.06%; net decrease of 0.76%) (Table 2). The proportion of individuals with hypertriglyceridemia, low HDL-cholesterol, and hyperglycemia increased in both genders. The proportion of individuals taking medications showed an increasing trend, suggesting that these problems have been receiving increased attention. (S1 Fig). Interestingly, the prevalence of individuals meeting the BP criterion decreased throughout the study periods regardless of gender, even though the proportion of individuals taking antihypertensives showed an increasing trend. This is in accordance with previous studies in the United States [12] and Korea [21] (S1 Fig).

**Table 2:** Proportion of subjects meeting the criteria for components of metabolic syndrome stratified by gender.

	Year					Percent change over the 4 years
	2009	2010	2011	2012	2013	
WC	29.39	28.95	29.26	28.95	29.63	0.24^a^
Men (>90cm) (%)	22.48	22.58	23.05	23.05	23.80	1.32 ^a^
Women (>80cm) (%)	35.82	34.88	35.05	34.44	35.06	-0.76 ^a^
TG (≥150mg/dL or medication use)	35.46	35.66	36.11	36.14	37.00	1.54 ^a^
Men (%)	43.65	43.77	44.44	44.33	44.99	1.34 ^a^
Women (%)	27.84	28.11	28.36	28.52	29.55	1.71 ^a^
HDL cholesterol	29.92	29.80	30.18	30.71	31.73	1.80 ^a^
Men (<40mg/dL or medication use) (%)	22.61	23.01	23.77	24.77	26.03	3.42 ^a^
Women (<50mg/dL or medication use) (%)	36.73	36.12	36.15	36.24	37.04	0.31 ^a^
Fasting glucose (≥100mg/dL or medication use)	32.88	32.53	32.65	32.86	34.10	1.22 ^a^
Men (%)	38.42	38.38	38.59	38.97	40.15	1.73 ^a^
Women (%)	27.73	27.09	27.13	27.18	28.47	0.74 ^a^
BP (≥130/85mmHg or medication use)	45.29	44.92	44.84	44.44	43.94	-1.35 ^a^
Men (%)	51.83	51.94	51.87	51.68	51.03	-0.80 ^a^
Women (%)	39.20	38.40	38.30	37.70	37.34	-1.86 ^a^

BP, blood pressure; HDL, high-density lipoprotein; TG, triglyceride; WC, waist circumference.

^a^General linear model was used for significance of trend from 2009 to 2013. *P* for trend < 0.01 for all comparison.

**Fig 3:**
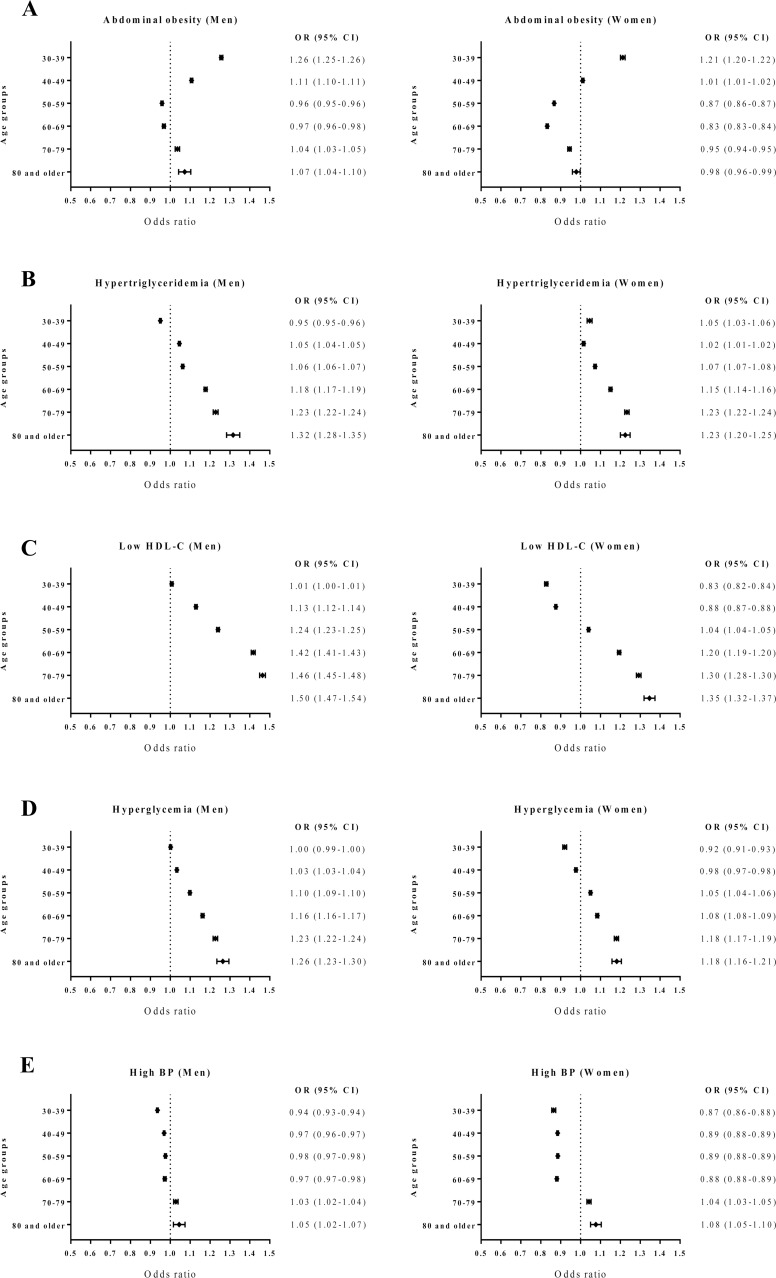
Forest plots of the ORs and CIs of components of metabolic syndrome (2013 versus 2009). BP = blood pressure, CI = confidence interval, HDL = high-density lipoprotein, OR = odds ratio.

Next, we stratified data according to age groups to reveal which age group accounted for the differences between the genders (Fig 3). Interestingly, women ≥50 years showed a significant decrease in abdominal obesity during the study period (Fig 3A). Although men aged 50−69 years also showed a decreasing trend, women in the same age groups exhibited a greater decrease. This decrease in abdominal obesity in middle-aged women may explain the decrease in abdominal obesity among women overall. On the other hand, men and women <50 years old showed an increased prevalence of abdominal obesity, which was augmented most in individuals 30−39 years old (OR = 1.26, 95% CI: 1.25, 1.26 for men; and OR = 1.21, 95% CI: 1.20, 1.22 for women). The prevalence of hypertriglyceridemia, low HDL-cholesterol, and hyperglycemia increased in both genders, especially in elderly individuals (Fig 3B–3D). By contrast, the prevalence of high BP significantly decreased in women and men of all age groups <70 years old, contributing to an overall decrease in the prevalence (Fig 3E).

The IFG has drawn special attention because it is associated with diverse metabolic outcomes including diabetes, CVD, and mortality [22–24]. We thus evaluated whether the presence of IFG affected the trends in prevalence of metabolic syndrome and its components. The prevalence of metabolic syndrome, defined as meeting two or more of the four criteria of abdominal obesity, hypertriglyceridemia, low HDL-cholesterol, and high BP, increased significantly over the study period in individuals with IFG (52.46% in 2009 and 54.50% in 2013; net increase of 2.03%). By contrast, the prevalence of metabolic syndrome did not increase in individuals with normal glucose metabolism (36.54% in 2009 and 36.48% in 2013) (Table 3). The prevalence of metabolic syndrome components except hypertension also increased significantly in the IFG group (Table 3).

**Table 3:** Prevalence of metabolic syndrome^*^ and its components in individuals with or without IFG.

	Year					Percent change over the 4 years
	2009	2010	2011	2012	2013	
Metabolic syndrome^a^						
Normal fasting glucose (%)	36.54	36.03	36.24	36.02	36.48	-0.06^b^
IFG (%)	52.46	52.80	53.63	53.71	54.50	2.03^b^
Abdominal obesity						
Normal fasting glucose (%)	25.76	25.24	25.46	25.06	25.47	-0.29^b^
IFG (%)	37.07	37.00	37.68	37.58	38.47	1.40^b^
TG (≥150 mg/dL or medication use)						
Normal fasting glucose (%)	30.81	30.82	31.11	31.04	31.63	0.82^b^
IFG (%)	44.52	45.27	46.18	46.28	47.20	2.68^b^
HDL-cholesterol						
Normal fasting glucose (%)	27.27	26.90	27.08	27.50	28.25	0.98^b^
IFG (%)	34.97	35.46	36.27	37.00	38.16	3.19^b^
BP (≥130/85 mmHg or medication use)						
Normal fasting glucose (%)	40.95	40.50	40.37	39.84	39.11	-1.85^b^
IFG (%)	54.04	54.08	54.11	53.95	53.40	-0.64^b^

BP, blood pressure; IFG, impaired fasting glucose; TG, triglyceride.

^a^Metabolic syndrome was defined as having two or more of the four components.

^b^General linear model was used for significance of trend from 2009 to 2013. *p* for trend <0.01 for all comparison.

## Discussion

In this large population-based health survey in Korea, we found that the prevalence of metabolic syndrome is increasing at an annual rate of 0.4%, even though the rate is lower than that of a previous Korean report (~0.7% per year [21]). In our study, the increase in prevalence was more prominent in men, and this gender difference was mainly attributed to a decreased prevalence of abdominal obesity in middle-aged women (age 50–69 years). A decreasing trend in obesity among Korean women was also reported in previous studies [25, 26], although the prevalence of obesity decreased in young but not middle-aged women [25, 26]. The authors suggested that an increased interest in leanness and increased proportion of the population participating in economic activity among young women may have contributed to a reduced prevalence of obesity [25]. Because the people who were lean at young ages are more likely to weigh less in their midlife [27], previous trends of weight loss in young Korean women may have exerted a similar association on the prevalence of abdominal obesity in middle-aged women in the current study. In addition, various campaigns through mass media and public institutions in recent years [28] may also have contributed to the decrease in abdominal obesity among middle-aged women.

However, the present study showed that the prevalence of abdominal obesity is increasing in young women and men in Korea. The cause of this discrepancy is unknown but may be related to a drastic increase in childhood obesity in Korea. Due to the socioeconomic development accompanying changes in eating habits, the prevalence of obesity in children and adolescents (6 to 17 years) has increased 4.6-fold in males and 3.2-fold in females from 1979 to 1996 [29], which may have exerted a detrimental effect on the prevalence of obesity among individuals aged 30−49 years in this study. Although recent studies in Korea showed that the prevalence of childhood obesity has stabilized since 2000 [30], continued public intervention to prevent childhood obesity is warranted.

The prevalence of hypertriglyceridemia and low HDL-cholesterol increased in both genders. These are significantly associated with lifestyle factors [31] such as alcohol consumption, reduced physical activity, and increased consumption of calories and simple sugars. In the present study, the number of heavy drinkers decreased significantly during the study period (Table 1). However, the mean total energy intake of Korean adults increased significantly (from 1883 kcal in 2009 to 2087 kcal in 2013) along with higher consumption of simple sugars [32]. Furthermore, the proportion of people who consume excess calories, defined as energy consumption greater than the estimated energy requirement from The Korean Nutrition Society, markedly increased from 33.7% in 2009 to 42.9% in 2013 [33]. Nonetheless, the proportion of individuals with experience in nutritional education decreased from 8.9% to 7.5% during the same study period [32]. Thus, active educational programs should be implemented to promote changes in dietary habits.

The proportion of individuals meeting high BP criteria decreased over the study period in both genders, despite an increase in metabolic syndrome. This has also been reported in a previous study [21], and may be related to increased public concern on hypertension and high salt intake. Indeed, a national survey performed in recent years reported decreased salt intake (4645 mg/day in 2009 and 4027 mg/day in 2013) and proportion of smokers (27.2% in 2009 and 24.1% in 2013).

Of particular interest in our study are the marked differences in trends among components of metabolic syndrome. Similar to what was reported previously [11, 12], we also observed sex and age differences in the prevalence of individual metabolic syndrome components. The decrease in the prevalence of hypertension and increase in that of IFG in our study are in line with findings previously reported in the United States [11]. On the other hand, the prevalence of dyslipidemia has decreased in the U.S. population [11], whereas it increased in our Korean population. The cause of this disparity among metabolic syndrome components and/or ethnicity is unclear to date, but our findings support previous suggestions [15, 16, 34] that not all components of metabolic syndrome are mediated by a single pathophysiologic process. For example, Meigs et al. [15] suggested that three distinct pathophysiological processes constitute metabolic syndrome, and that hypertension is not part of the “central metabolic syndrome” defined by high correlations among risk variables such as fasting and 2 hour plasma insulin, TG, HDL-cholesterol, waist-to-hip ratio (WHR), and BMI. Among these factors of “central metabolic syndrome,” hypertension was related to BMI but not with WHR, a factor well-known to represent central obesity and insulin resistance.

Measuring WHR is not easy in population-based studies, and WC has been considered the best simple index of abdominal visceral adiposity since the study by Pouliot et al. in 1994 [35]. Accordingly, a study in non-Hispanic whites and Mexican Americans reported that BMI and WC were as useful as WHR for identifying metabolic disorders [36]. On the other hand, studies in East Asia reported that WHR is a better predictor of fasting hyperglycemia or multiple metabolic risk factors than WC [37, 38], suggesting the possibility of ethnic differences. We did not measure WHR in this study thus we could not examine the contribution of WHR to the “central metabolic syndrome” components. However, our data showing disparate trends among the components suggested that not all components of metabolic syndrome can be grouped by a single etiologic factor [15, 16, 34]. To further support our contention, a recent study reported that hypertension was associated with both all-cause and CVD-related mortality, whereas type 2 diabetes mellitus was associated with CVD-related mortality only [39].

Of interest in our study was that the individuals with IFG, but not those with normal glucose metabolism, showed increasing prevalence of abdominal obesity, hypertriglyceridemia, and low HDL-cholesterol, which supports the concept that prediabetes is a key integrating mechanism of metabolic syndrome [40]. Given the trend for increased prevalence of other metabolic syndrome components in individuals with IFG, screening for metabolic syndrome should be considered in individuals recently diagnosed with IFG.

### Study limitations

Our study has some limitations that should be considered. First, we could not identify a causal relationship between metabolic syndrome and presumed etiologic factors due to the cross-sectional nature of the study. Second, the data may have recall bias because we used questionnaires to gather necessary information regarding participants’ history. Lastly, we could not follow the same individuals throughout the study periods due to the nature of repeated cross-sectional data. Despite these limitations, the large sample size (approximately 10 million individuals annually) is a major strength of our study and allowed us to accumulate the latest trend of metabolic syndrome in Korea with high statistical power.

## Conclusions

The prevalence of metabolic syndrome is still increasing significantly, further highlighting the deleterious effects of Westernized lifestyle and the inadequate efforts to decrease metabolic syndrome. Moreover, a remarkable increase in the prevalence of abdominal obesity in young adults emphasizes the importance of implementing lifestyle intervention programs to promote healthy dietary habits, physical activities, and better awareness of the risk of abdominal obesity in this particular age group. Considering the recent, rapid economic development and accompanying lifestyle changes that many countries have been experiencing, including South Korea, other developing or newly developed countries may also be encountering similar trends in metabolic syndrome to the one reported in this study. This kind of study on trends in the prevalence of metabolic syndrome might help in the design of better strategies for the prevention of metabolic syndrome pandemics.

## Supporting information

S1 Fig The prevalence of hypertriglyceridemia, hyperglycemia, and high blood pressure with the percentage of patients on medications. BP = blood pressure.
(TIF)
