# Supplementary figures and images for: Trends in the prevalence of metabolic syndrome and its components in South Korea: Findings from the Korean National Health Insurance Service Database (2009–2013)
Source: PLoS One. 2018 Mar 22;13(3):e0194490. doi: 10.1371/journal.pone.0194490 (PMC5864027; doi:10.1371/journal.pone.0194490)

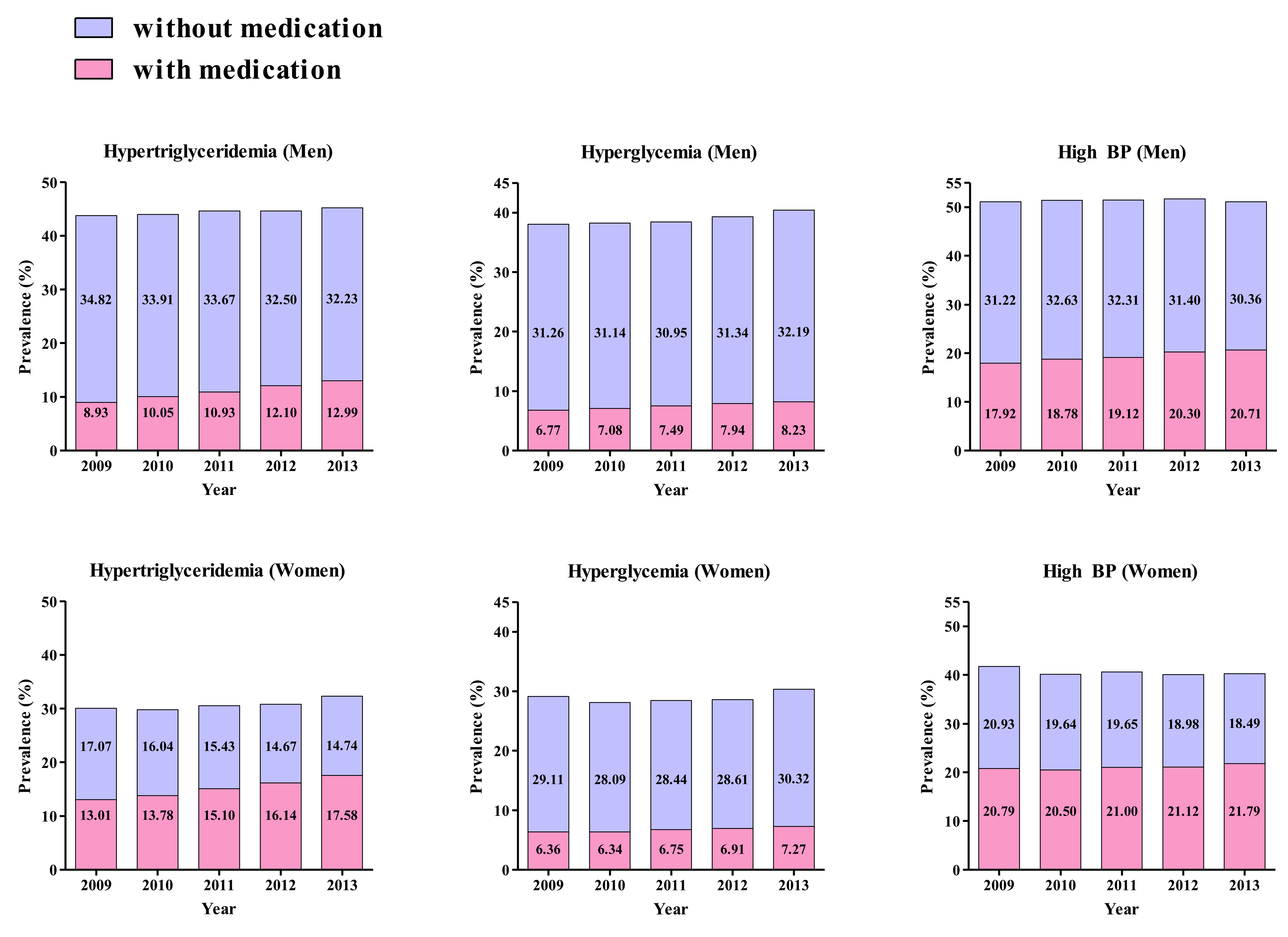

Supplement: S1 Fig — BP = blood pressure. (TIF) [file pone.0194490.s001.tif]
